# Irradiation of the potential cancer stem cell niches in the adult brain improves progression-free survival of patients with malignant glioma

**DOI:** 10.1186/1471-2407-10-384

**Published:** 2010-07-21

**Authors:** Patrick Evers, Percy P Lee, John DeMarco, Nzhde Agazaryan, James W Sayre, Michael Selch, Frank Pajonk

**Affiliations:** 1 Department of Radiation Oncology, David Geffen School of Medicine at UCLA, 10833 Le Conte Avenue, Los Angeles, CA-90095, USA; 2 Jonsson Comprehensive Cancer Center at UCLA, 10833 Le Conte Avenue, Los Angeles, CA-90095, USA; 3 UCLA School of Public Health, Biostatistics and Radiological Sciences, 10833 Le Conte Avenue, Los Angeles, CA-90095, USA

## Abstract

**Background:**

Glioblastoma is the most common brain tumor in adults. The mechanisms leading to glioblastoma are not well understood but animal studies support that inactivation of tumor suppressor genes in neural stem cells (NSC) is required and sufficient to induce glial cancers. This suggests that the NSC niches in the brain may harbor cancer stem cells (CSCs), Thus providing novel therapy targets. We hypothesize that higher radiation doses to these NSC niches improve patient survival by eradicating CSCs.

**Methods:**

55 adult patients with Grade 3 or Grade 4 glial cancer treated with radiotherapy at UCLA between February of 2003 and May of 2009 were included in this retrospective study. Using radiation planning software and patient radiological records, the SVZ and SGL were reconstructed for each of these patients and dosimetry data for these structures was calculated.

**Results:**

Using Kaplan-Meier analysis we show that patients whose bilateral subventricular zone (SVZ) received greater than the median SVZ dose (= 43 Gy) had a significant improvement in progression-free survival if compared to patients who received less than the median dose (15.0 vs 7.2 months PFS; P = 0.028). Furthermore, a mean dose >43 Gy to the bilateral SVZ yielded a hazard ratio of 0.73 (P = 0.019). Importantly, similarly analyzing total prescription dose failed to illustrate a statistically significant impact.

**Conclusions:**

Our study leads us to hypothesize that in glioma targeted radiotherapy of the stem cell niches in the adult brain could yield significant benefits over radiotherapy of the primary tumor mass alone and that damage caused by smaller fractions of radiation maybe less efficiently detected by the DNA repair mechanisms in CSCs.

## Background

Glioblastoma (GB) is the most aggressive form of brain tumors in adults. The current standard of care combines surgery and radiotherapy with drug treatment [1,2]. Even with this multi-modality approach, the median survival is only around 14 months with early recurrences primarily found in the brain.

Recent preclinical and clinical data provide convincing evidence that GBs are organized hierarchically with a small number of cancer stem cells (CSCs), which have the unique ability to self-renew and exhibit multi-lineage potency while their progeny lack these features [3-5]. The carcinogenesis of gliomas is still incompletely understood but carcinogenesis in general requires the acquisition of several mutations of tumor suppressor genes or oncogenes in normal cells to transform them into malignant tumor cells. In the human brain, the 3-5 mm thick lateral periventricular region of the lateral ventricles - the subventricular zone (SVZ) - and a subsection of the hippocampal formation - the subgranular layer (SGL) - have been shown to harbor normal brain stem cells [6-9]. These regions are believed to contain specific regions of so-called stem cell niches, which support neuronal stem cells and keep them in an undifferentiated state. A recent preclinical study suggested that tumor suppressor gene deletion in normal neural stem cells (NSCs) but not in their differentiated progeny leads to tumor formation in the brain [10]. It is suspected that specific niches in the brain also house CSCs [11] and that CSCs are relatively resistant to established anti-tumor therapies like radiation [12] and chemotherapy [13]. It has also been shown that the NSCs in these anatomical niches experience amplified division under conditions of cortical damage and their daughter cells (both glial and neuronal) are routed towards the point of injury, perhaps in a repair effort, as well as to the contralateral hemisphere [14]. Together, this provides a model by which we can understand how GB recurs bilaterally post-craniotomy as is often seen clinically [15].

According to the cancer stem cell hypothesis, all CSCs need to be eliminated in order to cure cancer [16]. Therefore, we hypothesized that the normal tissue stem cells niche in the brain may be a reservoir for CSCs from where they initiate and repopulate a tumor and that irradiation of the potential cancer stem cell niches in the brain would improve patient survival.

To test our hypothesis we performed a retrospective analysis of radiation treatment plans of high-grade glioma patients to study the effect of the radiation dose inadvertently delivered to the periventricular stem cells niche on the progression free survival (PFS) of these patients.

## Methods

### Patients

**Figure 1:**
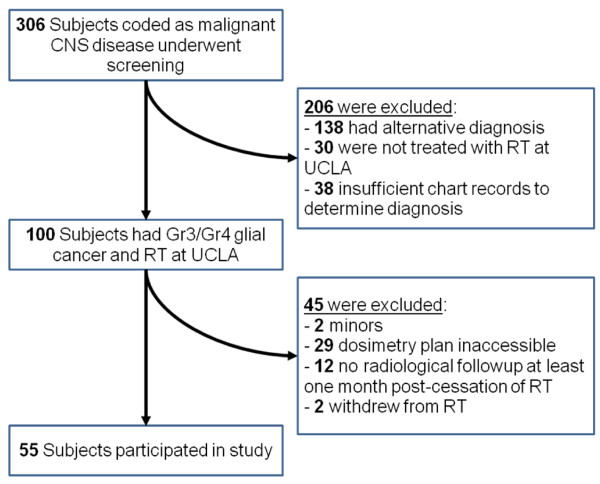
**Study subject selection process**. "Other" chemotherapeutics include CPT-11, Carboplatin, CCNU, and etoposide.

55 adult patients with histopathologically diagnosed grade 3 or grade 4 glial cancer who were treated using external beam radiation therapy at the University of California, Los Angeles (UCLA) between February of 2003 and May of 2009 were included in this retrospective study. The process of patient selection is outlined in Figure 1. Inclusion criteria were a histopathologically diagnosed anaplastic glioma (grade 3) or glioblastoma (grade 4), documented radiological follow-up for at least 1 month after cessation of radiotherapy, and accessible planning and dosimetry data within UCLAs Radiation Oncology records. Patients under the age of 18 and patients who failed to complete full radiotherapy treatment prescription were excluded from the study. This study was approved by the local Institutional Review Board at UCLA (IRB# 09-06-081-02).

### Contouring

**Figure 2:**
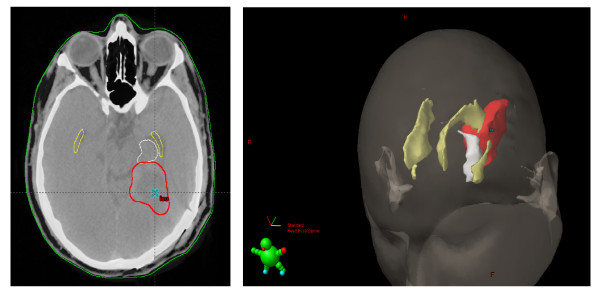
**The 2-dimentional CT contouring and resulting 3-dimensional reconstruction**. White: hippocampal formation. Yellow: periventricular region. Red: gross tumor volume. Reconstruction image anterosuperior view.

To capture the dosimetry associated with the subjects' NSC niches, this study required the contouring of the periventricular (PV) and hippocampal formation (HF) regions. Contouring the hippocampus was done in accordance with the protocol outlined by Chera et al [17]. Based on the operational definitions of the periventricular region, the SVZ was contoured as 3-5 mm lateral margin of the lateral ventricles [18] (Figure 2).

Due to the thin width of the periventricular contour, Eclipse's™ (Varian Medical Systems, Palo Alto, CA) post-processing modification functionality was necessary to extrapolate the inter-slice volumes and generate a continuous structure. The average volume of our reconstructed ipsilateral hippocampal formation was 4.4 cm^3 ^(95% CI: 4.0-4.8) and 5.05 cm^3 ^(95% CI: 4.69-5.40) for a unilateral periventricular region. Dose-volume histograms were calculated and mean dosing was extracted as the output of interest.

### PCR

DNA was isolated from FFPE using Ambion Recoverall Kit (Applied Biosystems/Ambion, Foster City, CA). DNA samples were bisulfite-modified using the Zymo Research EZ-DNA Methylation Gold Kit (Zymo Research, Orange, CA) according to manufacturer's directions. For MGMT, samples were subjected to a two-stage nested PCR strategy. The first-stage primers were 5'-GGATATGTTG GGATAGTT-3' and 5'-CCAAAAACCCCAAACCC-3' and second-stage primers were unmethylated reaction: 5'-TTTGTGTTTTGATGTTTGTAGGTTTTTGT-3' and 5'-AACTCCACACTCTTCCAAAAACAAAACA-3'; methylated reaction: 5'-TTTCGACGTTCGTAGGTTTTCGC-3' and 5'-GCACTCTTCCGAAAACGAAACG-3'. PCR products were analyzed on 3% agarose gels.

### Statistics

Statistical tests were executed SPSS (version 17; SPSS, Chicago, Illinois). The primary end-point in this retrospective study was PFS as defined as the number of months after the cessation of radiotherapy until the establishment of radiological evidence of disease progression. This establishment of radiological indication of progression must have been agreed upon by the treating neurooncologist.

The dosimetry data was extracted as a mean dose value for each structure of interest: ipsilateral PV, contralateral PV, bilateral PV, and ipsilateral HF. For each structure, these doses were divided at their median value into a high dose arm (those patients who received greater than the median dose) and a low dose arm (those patients who received a less than median dose).

Kaplan-Meier plots were calculated with event times being the time since the cessation of therapy until progression or death, whichever occurred first [19]. Subjects who neither died nor had cancer progressing were censored at time of their last follow-up. Patients were stratified based the total prescribed dose and dose delivered to both periventricular regions.

Relative risks were calculated using the Cox proportional hazards regression model [20]. Dose to the bilateral periventricular region was entered as a continuous variable. A model including dose to the periventricular region, total dose administered, RPA (recursive partitioning analysis) class, total resection intervention, and tumor location as construct model effects provided adjusted relative risks that corresponded to the Kaplan-Meier analyses. The log partial likelihood was compared with that of a model without interaction in order to test whether the dose to the periventricular region relative risks differed significantly with between the low- and the high-dose group. The log partial likelihood ratio test was used.

## Results

The patient population was divided along the median bilateral periventricular dose (= 43 Gy) into a high dose group containing 28 subjects, and a low dose group containing 27 subjects. Table 1 illustrates that both groups were relatively well balanced for the parameters assessed, except for gender.

**Table 1:** Study subject characteristics

		# of Subjects	
		Low PV Dose	High PV Dose
**Subjects**		27	28
**Gender**	Male	18	12
	Female	9	16
**Mean Age at RT (range)**		52 ± 6 (25-82)	51 ± 5 (26-77)
**Glioma Grade**	Grade 3	8	9
	Grade 4	19	19
**Racial-Ethnicity**	Caucasian	25	26
	Asian	2	2
**Surgical Co-Intervention**	Biopsy-only	2	5
	Total Resection	16	6
	Subtotal Resection	9	17
**Drug Co-intervention**	Temodar	27	27
	Avastin	5	9
	Other	8	3
**Median Karnofsky Perf. Scale (range)**		90% (70-100%)	90% (50-90%)
**Mean RPA Classification**	# of RPA Class III	8	8
	# of RPA Class IV	11	8
	# of RPA Class V	8	12
**MGMT Status**	Methylated	2	6
	Unmethylated	5	6
	Unknown	20	16
**Tumor Localization**	Frontal	6	11
	Temporal	12	10
	Parietal	4	3
	Occipital	2	1
	Other	3	3
**Mean Bilateral PV Dose**		27 Gy ± 5	50 Gy ± 2
**Median Ipsilateral PV Dose**		46 Gy ± 15.5	
**Median Contralateral PV Dose**		41 Gy ± 16.1	
**Median Ipsilateral HF Dose**		49.9 Gy ± 16.3	
**Mean Total Prescription Dose (range)**		52 Gy ± 4 (30-63)	57 Gy ± 2 (45-60)

**Figure 3:**
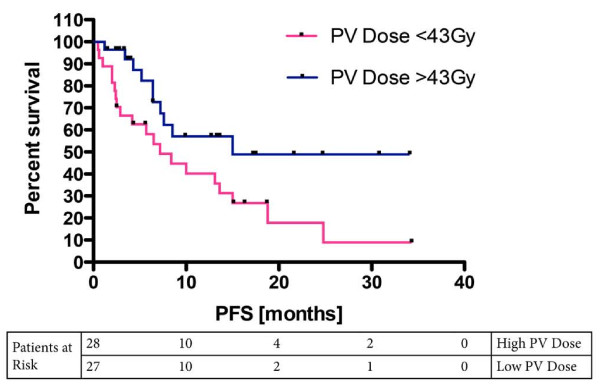
**Kaplan-Meier survival curve illustrating the progression-free survival difference between those subjects that received high-dose RT and low-dose RT to their periventricular regions**. p < 0.05, log-rank test.

Of the 55 subjects evaluated, the median dose to the bilateral PV was 43 Gy while that to the HF was 47 Gy. A Kaplan-Meier analysis of the PV dosing illustrated that the survival curve for those receiving greater than the median dose of 43 Gy was statistically significantly different than those who received less than the median dose (Figure 3). The median PFS for the high PV dose group was 15 months while the median PFS for the low PV dose group was 7.2 (p = 0.03, log-rank test). Two similar Kaplan-Meier analyses of the ipsilateral HF dosing, and ipsilateral PV dosing alone did not yield statistically significant results.

**Figure 4:**
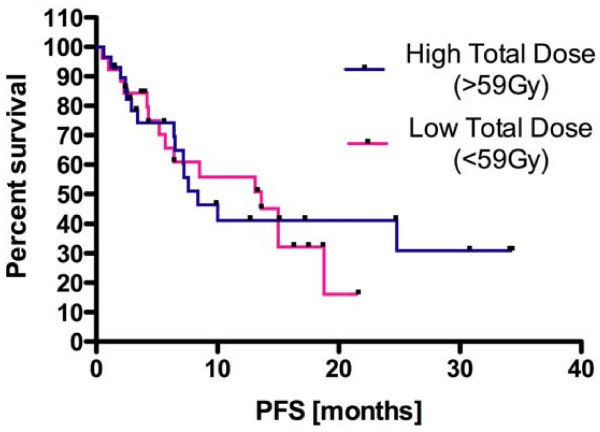
**Kaplan-Meier survival curve illustrating the progression-free survival difference between those subjects that received more or less than the median prescription dose did not yield statistically significant results**. p = 0.84, log-rank test.

To assess the simple impact of prescription dose on PFS in this patient pool, a Kaplan-Meier analysis in which patients were divided by the median total prescribed dose of 59.4 Gy was analyzed for progression-free survival, did not yield statistically significant results (p = 0.83, log-rank test, Figure 4). Likewise, a correlation analysis between total prescribed dose and bilateral PV dosing showed only very weak correlation (R^2 ^= 0.1).

To further quantify the impact of bilateral PV periventricular dosing while controlling for various demographic characteristics, a Cox regression analysis was performed. Table 2 presents the results of this analysis that includes all patients and models the effects of dose to the periventricular region, location of the tumor, surgical intervention, RPA classification, and the total prescribed tumor dose as constants over time. Radiation dose given to the periventricular regions was associated with decreased risk of progression for patients (relative risk: 0.74; P = 0.019, 95% CI: 0.567 - 0.951, Cox partial likelihood ratio test) while the risk ratios for all other parameters did not reach statistical significance.

**Table 2:** Cox regression analysis of progression-free survival

Parameter	*Risk Ratio*	*Lower CL*	*Upper CL*	*P*	*Variable Type*
**Tumor location**	1.016	0.77	1.341	0.908	categorical
**RPA Class**	1.233	0.689	2.206	0.481	categorical
**Tumor Resection**	0.546	0.228	1.308	0.546	categorical
**Periventricular Dose**	0.735	0.567	0.951	0.019	continuous
**Total Tumor Dose**	0.794	.451	1.396	0.423	continuous

## Discussion

Even after optimal conventional treatment, the outcome for patients suffering from GB is currently unacceptable and drugs like temozolamide add only little survival benefit for the patients, which comes at very high costs and is accompanied by considerable toxicity [1]. Presently, while many other cancers are cured by radiation treatment, most patients suffering from glioblastoma relapse after radiotherapy. This is despite the fact that patients suffering from glioblastoma are generally treated with relatively high total radiation doses and SF_2 Gy _(surviving fraction at 2 Gy) values for glioblastoma cells in-vitro are not exceptionally high [21]. A possible explanation for the failure of radiotherapy to cure patients from GB is the observation that glioma cells migrate widely into healthy bilateral brain tissue [22,23] from one or more foci of origin. These isolated cells are not detected by current radiological techniques or even biological imaging and therefore usually not included into the target volume during radiotherapy.

In our present study we tested the hypothesis that the dose prescribed to the normal tissue stem cell niche in the adult brain would impact the effectiveness of radiotherapy for patients suffering from glioblastoma as these niches may serve as a harbor for radioresistant glioma stem cells, which are the only cells in a glioblastoma believed to able to repopulate a tumor [16]. Our hypothesis was based on previous reports showing that adult normal tissue stem cells reside in the lateral periventricular regions of the lateral ventricles and the hippocampus [6,9] and animal studies reporting that transformation of normal tissues stem cells but not differentiated cells lead to tumor formation [24-27]. The unique anatomical patterns of the brain that clearly separate stem cell niches as a potential pool of CSCs from differentiated tissue make the brain an ideal model system to study the impact of radiation dose given to these stem cell niches.

**Figure 5:**
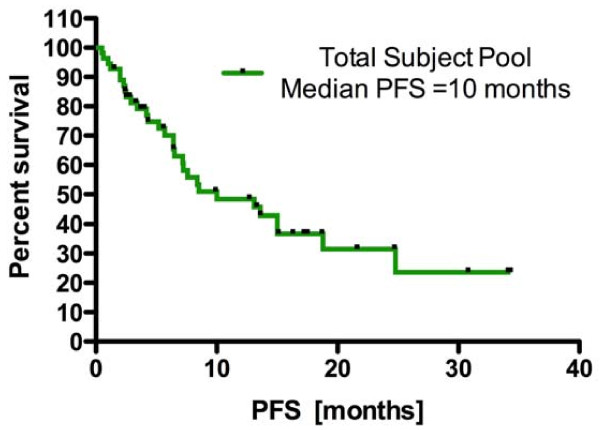
**Kaplan-Meier curve reflecting the overall progression-free survival of all patients**.

All but one (54/55) of our patients received radiotherapy and temozolamide. The mean PFS was 9.6 months (95% CI: 7.2 - 11.9, Figure 5). We analyzed the PV as a single bilateral structure given the literature illustrating that neural stem cells can seed daughter cells along the distribution pattern of callosal fibers into the contralateral and ipsilateral cortical hemispheres [14,28]. This process is particularly active in response to cortical injury, as we would see in conditions of GB. Here we report that the mean radiation dose applied to both periventricular regions during fractionated radiotherapy was correlated with a 108% increase in the median PFS of patients suffering from glioma (7.2 vs. 15.0 months, p = 0.03, log-rank test). The mean dose applied to neither the ipsilateral and contralateral PV region alone nor the mean dose delivered to the ipsilateral hippocampal region was correlated with prolonged PFS. The failure of total prescription dose to render statistical significance in the Cox proportional hazard model as well as its weak coefficient of determination with PV dosing excluded prescription dose applied to the tumor volume as a possible explanation of our data.

Our study has several shortcomings. First, this is a retrospective analysis and as such, it needs validation in a prospective trial. Second, even though all but one patient received temozolamide in combination with radiation, we do not have MGMT (O6-methylguanine-DNA-methyltransferase) promoter methylation data for all our patients. Therefore, it is possible that that the group of patients receiving higher doses to the PV region had predominantly unmethylated MGMT promoters. Finally, we did not assess the effect of the PV dose on overall survival because after tumor recurrence patients were subjected to various salvage therapies. The heterogeneous nature of the treatments applied would not have allowed for any conclusions regarding the radiation treatment and future studies would have to account for this in the study design.

However, our observation fits well into the current understanding of neural progenitor cell migratory patterns out of these stem cell niches. In the adult brain, the SGL NSCs send their differentiated daughter cells solely into the granular layer of the hippocampus. No evidence for the SGL distributing cells throughout the cortex has yet been described. Accordingly, our data indicating that the magnitude of radiotherapy directed at the SGL provides no significant impact on progression is unsurprising. The periventricular SVZ however, has been shown by Goings et al. to respond to cortical injury by increasing neurogenesis and sending migratory cells into the corpus callosum, in the cortex at the edge of the lesion and on migratory projections between the lesion and the SVZ [14]. Incorporating this migratory pattern with the assertions of Llaguno et al. that it is the NSCs held in the SVZ that hold the malignant transformation potential to give rise to glioma [10], our data indicate that efforts to eradicate these SVZ CSCs bilaterally may lead to a survival advantage for glioblastoma patients.

Previous clinical studies prescribing 45 Gy to the normal brain with a 15 Gy boost for the tumor region did not yield in significant improvements of clinical outcome [29]. The major differences between these studies and our analysis is that the SVZ in our study was mostly outside the clinical target volume and radiation applied to the SVZ was therefore given in smaller fractions (1.36 Gy, CI: 1.2 - 1.5). Additionally, radiotherapy techniques and imaging capabilities have dramatically evolved since these early trials and therefore, these historic data may not easily be comparable to modern treatment. The relative resistance of CSCs to ionizing radiation is believed to be caused by increased free radical scavenger expression [30] and activation of DNA damage checkpoints [12] in CSCs. We hypothesize that CSCs exhibit a threshold for DNA damage recognition and damage caused by subclinical fractions of radiation may remain undetected by the DNA repair response in CSCs. This is supported by previous reports describing low dose hypersensitivity for GB cells [31,32] although low-dose hypersensitivity has not been studied in CSCs specifically yet and needs to be addressed in future studies.

## Conclusions

In this study, we have illustrated an increase in PFS among glioblastoma patients when treated with periventricular dosing in excess of 43 Gy. Prescription of a defined dose to the periventricular region may be a way to improve treatment responses in patients with high-grade gliomas. However, the periventricular regions and the hippocampus are locations of adult neurogenesis and thought to contribute to normal tissue repair in the brain. Even though current survival times for most GB patients are too short to allow the observation of long-term normal tissue side effects this may change with improved outcome. Therefore, prospective, randomized clinical trials should be initiated to address the efficacy and toxicity of including the periventricular regions as additional target volumes into treatment plans for patients suffering from glioblastoma with optimal dosing yet to be determined.

## Conflict of interests

The authors declare that they have no competing interests.

## Authors' contributions

'PE analyzed, interpreted the data, and drafted the manuscript, PPL helped designing the study and drafting the manuscript, JD and NA analyzed the data and helped drafting the manuscript, JWS performed the statistical analysis and helped drafting the manuscript, MS helped drafting the manuscript, FP conceived of the study, analyzed and interpreted the data, and drafted the manuscript. All authors read and approved the final manuscript.

## Pre-publication history

The pre-publication history for this paper can be accessed here:

http://www.biomedcentral.com/1471-2407/10/384/prepub
